# Diffusion MRI quantifies early axonal loss in the presence of nerve swelling

**DOI:** 10.1186/s12974-017-0852-3

**Published:** 2017-04-07

**Authors:** Tsen-Hsuan Lin, Chia-Wen Chiang, Carlos J. Perez-Torres, Peng Sun, Michael Wallendorf, Robert E. Schmidt, Anne H. Cross, Sheng-Kwei Song

**Affiliations:** 1 Radiology, Washington University School of Medicine, 660 S Euclid Ave, St. Louis, MO 63110 USA; 2 Biostatistics, Washington University School of Medicine, 660 S Euclid Ave, St. Louis, MO 63110 USA; 3 Pathology, Washington University School of Medicine, 660 S Euclid Ave, St. Louis, MO 63110 USA; 4 Neurology, Washington University School of Medicine, 660 S Euclid Ave, St. Louis, MO 63110 USA; 5 Hope Center for Neurological Disorders, Washington University School of Medicine, 660 S Euclid Ave, St. Louis, MO 63110 USA; 6 Biomedical Engineering, Washington University, 1 Brookings Dr, St. Louis, MO 63130 USA; 7 Current Address: Institute of Biomedical Engineering and Nanomedicine, National Health Research Institute, 35 Keyan Road, Zhunan, Miaoli County 35053 Taiwan; 8 Current Address: School of Health Sciences, Purdue University, 550 W Stadium Ave, West Lafayette, IN 47907 USA; 9 Biomedical MR Laboratory, Washington University School of Medicine, Campus Box 8227, Room 2313, 4525 Scott Ave, St Louis, MO 63110 USA

**Keywords:** Optic neuritis, Multiple sclerosis, Axonal loss, DBSI, Diffusion MRI

## Abstract

**Background:**

Magnetic resonance imaging markers have been widely used to detect and quantify white matter pathologies in multiple sclerosis. We have recently developed a diffusion basis spectrum imaging (DBSI) to distinguish and quantify co-existing axonal injury, demyelination, and inflammation in multiple sclerosis patients and animal models. It could serve as a longitudinal marker for axonal loss, a primary cause of permanent neurological impairments and disease progression.

**Methods:**

Eight 10-week-old female C57BL/6 mice underwent optic nerve DBSI, followed by a week-long recuperation prior to active immunization for experimental autoimmune encephalomyelitis (EAE). Visual acuity of all mice was assessed daily. Longitudinal DBSI was performed in mouse optic nerves at baseline (naïve, before immunization), before, during, and after the onset of optic neuritis. Tissues were perfusion fixed after final in vivo scans. The correlation between DBSI detected pathologies and corresponding immunohistochemistry markers was quantitatively assessed.

**Results:**

In this cohort of EAE mice, monocular vision impairment occurred in all animals. In vivo DBSI detected, differentiated, and quantified optic nerve inflammation, demyelination, and axonal injury/loss, correlating nerve pathologies with visual acuity at different time points of acute optic neuritis. DBSI quantified, in the presence of optic nerve swelling, ~15% axonal loss at the onset of optic neuritis in EAE mice.

**Conclusions:**

Our findings support the notion that axonal loss could occur early in EAE mice. DBSI detected pathologies in the posterior visual pathway unreachable by optical coherence tomography and without confounding inflammation induced optic nerve swelling. DBSI could thus decipher the interrelationship among various pathological components and the role each plays in disease progression. Quantification of the rate of axonal loss could potentially serve as the biomarker to predict treatment outcome and to determine when progressive disease starts.

## Background

Multiple sclerosis (MS) is an inflammatory demyelinating disease producing, ultimately, irreversible axonal loss and permanent neurological impairments [1–4]. The axonal pathology is complex with components directly associated with the fiber tracts (axonal injury/loss and demyelination) and those surrounding the tracts (immune cell infiltration and edema). Each of these axonal pathology components may contribute to neurological dysfunction and therefore to the clinical signs and symptoms of MS [4–6]. Although inflammation and demyelination each contributes to MS pathophysiology, axonal loss is believed to be the primary correlate of irreversible neurological disability [7, 8]. Therefore, the development of a non-invasive biomarker to reflect the extent of axonal loss and the severity of damage in surviving axons is paramount to confirmation of this notion, to better monitor individual patients, and to use as an endpoint in trials of potential therapeutics. Optic neuritis is commonly one of the first manifestations of MS [9, 10]. Optic neuritis, much like MS, is characterized by inflammatory demyelination and varying degrees of axonal injury [10]. Optic nerve dysfunction leads to impairment of visual function which can be monitored in mice in a clinically relevant manner [11]. As such, mouse models of optic neuritis present an opportunity to evaluate the connection between imaging, pathology, and function in a disorder like MS.

Several different magnetic resonance imaging (MRI) biomarkers have recently been evaluated in MS [12–14]. Diffusion tensor imaging (DTI), in particular, is one of the commonest tools for evaluating white matter disease as, under some circumstances, it can distinguish axonal injury from demyelination [15–17]. However DTI-derived metrics are obfuscated by the presence of inflammatory pathology [18, 19]. We recently developed a new diffusion MRI approach called diffusion basis spectrum imaging (DBSI) that is able to separately quantify the axonal and inflammatory pathologies [20, 21]. DBSI models the diffusion signal as a linear combination of anisotropic diffusion tensors reflecting fibers, which in white matter are predominantly axon fibers, and a spectrum of isotropic diffusion tensors which encompass cells, edema, and cerebrospinal fluid [20, 22]. In the study reported here, we applied DBSI at the onset of optic neuritis (ON) in the experimental autoimmune encephalomyelitis (EAE) mouse model. DBSI is able to distinguish and quantify axon injury, demyelination, cellular infiltration and edema, and axonal loss.

## Methods

### EAE mouse model of optic neuritis

All experiments were performed on 10-week-old female C57BL/6 mice (The Jackson Laboratory, Bar Harbor, ME). All mice were housed and maintained in the Washington University animal facility and subjected to a 12-h light/dark cycle with constant access to nourishments. The EAE model of optic neuritis was induced as previously described [22]. Mice were immunized with 50 μg myelin oligodendrocyte peptide (MOG_35-55_) emulsified in incomplete Freund’s adjuvant with 50 μg *Mycobacterium tuberculosis*. Mice further received 300 ng intravenous adjuvant pertussis toxin (PTX, List Laboratories, Campbell, CA) on the day of and 2 days after immunization. Eight mice were studied.

### Visual acuity (VA) measurements

Visual acuity, utilized to measure visual function in parallel to clinical signs, was assessed with the Virtual Optometry System (Optomotry, Cerebral Mechanics, Inc., Canada) as previously described [23]. In short, mice were presented with virtual rotating columns displayed on four LCD screens. The spatial frequencies in cycle/degree (c/d) were changed starting from 0.1 c/d with step size of 0.05 c/d until the mouse stopped responding. VA is then defined as the highest spatial frequency to which the mouse was able to respond. Left and right eye VA can be assessed by the direction of rotating columns, clockwise for left eye and vice versa [24]. If the mouse did not respond to 0.1 c/d, VA was assigned to be 0 c/d. With this technique, it is possible to separately assess the VA of each eye by switching the rotational direction of the columns. Visual impairment was defined as VA ≤0.25 c/d, based on our previous work [22]. Normal VA was confirmed before immunization and then assessed daily after immunization.

**Fig. 1:**
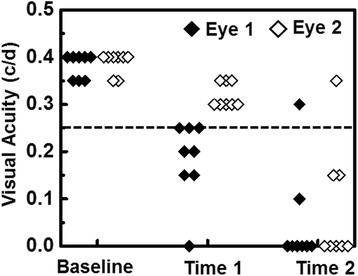
Visual acuity (VA) of EAE mice (*n* = 8) from baseline, pre-onset (time 1 of eye 2), onset (time 1 of eye 1 and time 2 of eye 2), and post-onset (time 2 of eye 1). Eye 1 = *filled symbols*; eye 2 = *open symbols*. The *dotted line* indicates VA = 0.25 c/d, which was the threshold of defined onset of optic neuritis. The results indicated that there was no visual acuity difference between eye 1 and eye 2 at time 2 (*p* = 0.15)

For each mouse in our cohort, onset of optic neuritis, as indicated by impairment of visual function defined by VA, did not occur simultaneously for both eyes. We therefore defined time 1 as the day in which the first eye had a VA ≤0.25 c/d and time 2 as the day in which the second eye had a VA ≤0.25 c/d. Concordantly, for each mouse, eye 1 is the eye affected at time 1 and eye 2 is the eye affected at time 2. VA for each eye is presented in Fig. 1. Based on this experimental paradigm, time 1 and time 2 corresponded to onset and post-onset of optic neuritis for eye 1 and pre-onset and onset of optic neuritis for eye 2, respectively.

### Magnetic resonance imaging (MRI) measurements

MRI experiments were performed on a 4.7 T Agilent DirectDrive™ small-animal MRI system (Agilent Technologies, Santa Clara, CA) equipped with Magnex/Agilent HD imaging gradient coil (Magnex/Agilent, Oxford, UK) with pulse gradient strength up to 58 G/cm and a gradient rise time ≤295 μs. Mice were anesthetized with 1% isoflurane in oxygen and placed in a custom made 3-point immobilization head holder. Breathing rate was monitored, and body temperature was maintained at 37 °C with a small animal physiological monitoring and control unit (SA Instruments, Stony Brook, NY). An actively decoupled volume (transmit)/surface (receive) coil pair was used for MRI excitation and signal reception. Diffusion-weighted MRI data was acquired with a transverse slice of mouse brain with two optic nerves, as nearly orthogonal to the image slice as possible. A multi-echo spin-echo diffusion-weighted sequence [25] with an icosahedral 25-direction diffusion-encoding scheme [26] combined with one *b* = 0 was employed and MR acquisition parameters were TR of 1.5 s, TE of 37 ms, time between gradient pulses (Δ) of 18 ms, gradient pulse duration (δ) of 6 ms, maximum *b*-value of 2200 s/mm^2^ (each encoding direction has a unique *b*-value), slice thickness of 0.8 mm, and in-plane resolution of 117 μm^2^.

### MRI data analysis

Data was analyzed with DBSI multi-tensor and conventional DTI single-tensor analysis packages developed in-house with Matlab [20, 21]. For optic nerve, we have a coherent fiber bundle, the diffusion-weighted imaging data was modeled according to Eq. 1:

1 $$ {S}_k= f{e}^{-\left|\overset{\rightharpoonup }{b_k}\right|{\lambda}_{\perp }}{e}^{-\left|\overset{\rightharpoonup }{b_k}\right|\left({\lambda}_{\parallel }-{\lambda}_{\perp}\right){ \cos}^2{\Phi}_k}+{\displaystyle {\int}_a^b f(D){e}^{-\left|\overset{\rightharpoonup }{b_k}\right| D} dD\kern1em \left( k=1,\ 2,\ 3,\dots,\ 25\right)}. $$

The quantities *S*_*k*_ and $\left|\overset{\rightharpoonup }{b_k}\right|$ are the signal and *b*-value of the *k* ^*th*^ diffusion gradient, Φ_*k*_ is the angle between the *k* ^*th*^ diffusion gradient and the principal direction of the anisotropic tensor, *λ*_||_ and *λ*_⊥_ are the axial and radial diffusivities of the anisotropic tensor, *f* is the signal intensity fraction for the anisotropic tensor, and *a* and *b* are the low and high diffusivity limits for the isotropic diffusion spectrum (reflecting cellularity and edema, respectively) *f*(*D*). DBSI derived *f* represents retinal ganglion cell (RGC) axon density (fiber fraction) in the image voxel, accounting for intra-voxel pathological and structural complications. DBSI derived λ_||_ and λ_⊥_ reflect residual axon and myelin integrity respectively: ↓ λ_||_ ≈ axonal injury and ↑ λ_⊥_ ≈ demyelination [20–22, 27]. Based on our previous experimental findings, the restricted isotropic diffusion fraction reflecting cellularity is derived by the summation of *f*(*D*) at 0 ≤ ADC ≤ 0.3 μm^2^/ms. The summation of the remaining *f*(*D*) at 0.3 < ADC ≤ 3 μm^2^/ms represents non-restricted isotropic diffusion, which putatively denotes vasogenic edema and CSF [20–22, 27].

Regions of interest (ROI) were manually drawn in the center of each optic nerve on the diffusion-weighted image, corresponding to the diffusion gradient direction perpendicular to optic nerves, to minimize partial volume effects. ROIs were then transferred to the parametric maps to calculate the mean for each of the DBSI and DTI-derived metrics.

### ROI for DBSI fiber fraction

Separate ROIs encompassing the whole optic nerve were drawn on the diffusion-weighted images (DWI) with diffusion-weighting gradient orthogonal to the optic nerve, which were larger than the ROIs for measuring DBSI- or DTI-derived metrics. The partial volume effect of surrounding cerebrospinal fluid for ROIs outlining optic nerve cross-section area estimation was minimized because surrounding cerebrospinal fluid signal was eliminated via diffusion weighting. DBSI analysis models axonal fibers as anisotropic diffusion tensor components excluding any residual free isotropic CSF signal.

### Immunohistochemistry

Immediately after the final MRI time point, mice were deeply anesthetized and underwent perfusion via the left cardiac ventricle with phosphate-buffered saline (PBS) followed by 4% paraformaldehyde (PFA). Brains were excised after intra-cardiac perfusion fixation with 4% PFA at 4 °C and then transferred to PBS for further storage until processing. Optic nerves were then dissected, embedded in 2% agar, and then further embedded in paraffin wax [28]. Paraffin blocks were sectioned at 5-μm thick, deparaffinized, and rehydrated for immunohistochemistry analysis. Sections were blocked with 5% normal goat serum and 1% bovine serum albumin in PBS for 30 min at room temperature to prevent non-specific binding. Slides were then incubated overnight at 4 °C with primary antibody and then 1 h at room temperature with the appropriate secondary antibody. Primary antibodies used were anti-total neurofilament (SMI-312, BioLegend, 1:300), anti-phosphorylated neurofilament (SMI-31, BioLegend, 1:300), and anti-myelin basic protein (MBP, Sigma, 1:300). Secondary antibodies were goat anti-mouse or goat anti-rabbit (Invitrogen, 1:240) with both conjugated to Alexa 488. Slides were mounted with Vectashield Mounting Medium for DAPI (Vector Laboratory, Inc., Burlingame, CA) and coverslipped. Images were acquired on a Nikon Eclipse 80i fluorescence microscope with MetaMorph software (Universal Imaging Corporation, Sunnyvale, CA) at ×72 and ×84 (1.2 and 1.4 magnification of ×60 objective) magnifications. Quantification was performed on entire optic nerve images which were the combination of four to six ×72 immunohistochemistry images using ImageJ (http://rsbweb.nih.gov/ij/plugins/volume-viewer.html, NIH, US). Images were then undergone background subtraction, bilateral filter for edge preservation, watershed segmentation, threshold determination, and the analyze particles macro for SMI-312, SMI-31, and MBP area calculation and then normalized by entire area of optic nerve. Background subtraction, watershed segmentation, threshold determination, and analyze particles were used for DAPI counts.

### Statistics

For all the boxplots, whiskers extend to the minimum/maximum and the mean is marked as diamonds. Data were collected in a nested design where each mouse had two periods (eye 1 and 2) each containing repeated measurements at baseline, time 1, and time 2. Data were analyzed with a mixed random effect repeated measures model with period, time, and period by time interaction fixed effects. Contrasts were estimated for change from baseline to times 1 and 2, averaged over periods. Degrees of freedom were adjusted with Kenward-Rogers method. A first order auto-regressive covariance structure was used to account for repeated measures. The correlation of histology data and DBSI measurements at time 2 were analyzed by simple linear regression.

## Results

### Monocular visual acuity decrease at the onset of optic neuritis

After immunization, daily VA of EAE mice (*n* = 8) was confirmed. When VA ≤ 0.25 c/d, defined as onset of ON [22], DBSI was performed and the eye was defined as eye 1 at time 1 (12.1 ± 1.9 days post-immunization, mean ± SD, *n* = 8). The other eye was defined as eye 2. When the VA of eye 2 decreased below 0.25 c/d, DBSI was performed again at the same day (14.4 ± 1.7 days post-immunization, mean ± SD, *n* = 8, one eye 2 did not develop ON but still included in statistical analyses) defined as time 2 (Fig. 1). There was no difference between eye 1 and eye 2 at time 2 (*p* = 0.15).

### DBSI reflected acute inflammation and axonal pathology specifically

**Fig. 2:**
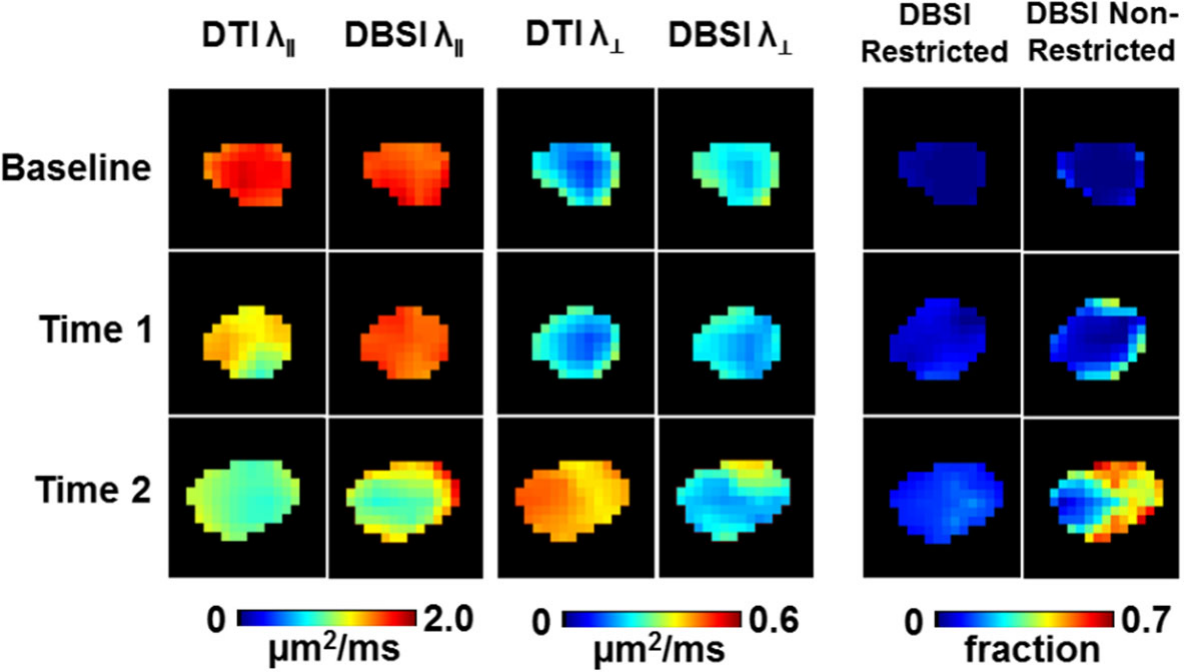
DTI- and DBSI-derived parametric maps of one representative EAE optic nerve (eye 1) from baseline (*top row*), time 1 (*middle row*, onset), and time 2 (*bottom row*, onset of the fellow eye). Decreased axial diffusivity (λ_ǁ_, *columns 1 and 2*) and increased radial diffusivity (λ_⊥_, *columns 3 and 4*) in both DTI and DBSI measurements suggest axonal injury and demyelination at time 2. DBSI distinguished and further quantified the extent of inflammatory cell infiltration (*column 5*, restricted diffusion fraction) and vasogenic edema (column 6, non-restricted diffusion fraction), confounding DTI estimated λ_ǁ_ and λ_⊥_

**Fig. 3:**
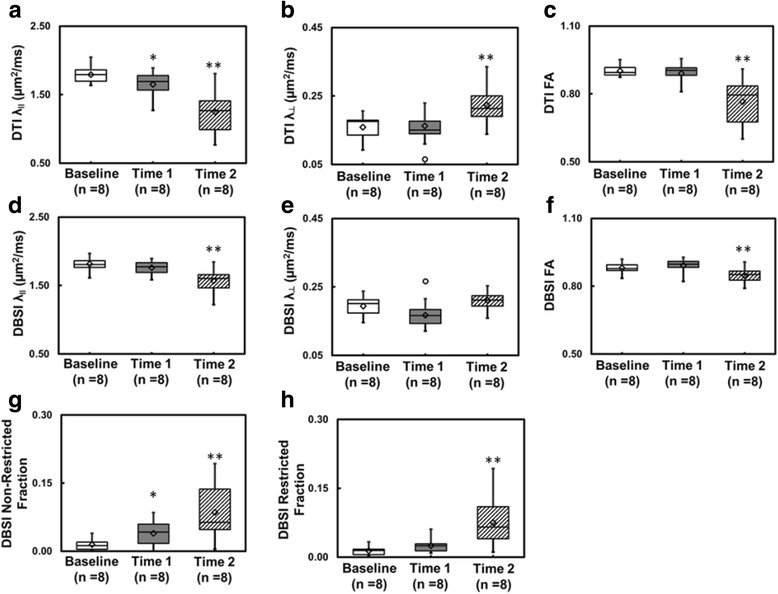
Box plots summarize the group distribution of DTI-derived λ_ǁ_, λ_⊥_, and FA (**a**–**c**) and DBSI-derived λ_ǁ_, λ_⊥_, FA, restricted, and non-restricted diffusion fraction (**d**–**h**) from baseline, time 1, and time 2, respectively. Axonal injury developed at time 2 suggested by the significantly decreased DTI- and DBSI-λ_ǁ_ (**a**, **d**, *p* < 0.005). At time 1, significant decrease was only seen in DTI-λ_ǁ_ but not in DBSI-λ_ǁ_ reflecting the confounding effects of inflammatory cell infiltration and vasogenic edema (**a**, **d**). The same confounding effects also resulted in increased DTI-λ_⊥_ at time 2 but not in DBSI-λ_⊥_ (**b**, **e**).The distribution of DBSI results (**d**–**f**) was much tighter than DTI (**a–c**) since DBSI was able to separate vasogenic edema (**g**) and cell infiltration (**h**) from axon and myelin pathologies. *One asterisk* indicates *p* < 0.05. *Double asterisks* indicate *p* < 0.005

DTI- and DBSI-derived parametric maps from EAE optic nerve revealed decreased DTI-λ_∥_ (1.65 ± 0.16 μm^2^/ms vs. control 1.79 ± 0.12 μm^2^/ms, *p* < 0.05), normal DBSI-λ_∥_ (1.82 ± 0.08 μm^2^/ms vs. control 1.79 ± 0.12 μm^2^/ms, *p* = 0.059) and normal DTI- λ_⊥_(0.16 ± 0.03 μm^2^/ms vs. control 0.16 ± 0.04 μm^2^/ms, *p* = 0.088) and DBSI-λ_⊥_ (0.20 ± 0.03 μm^2^/ms vs. control 0.17 ± 0.03 μm^2^/ms, *p* = 0.065) at time 1 (Fig. 2). Similarly, mild but not significant inflammatory cell infiltration and putative significant vasogenic edema, manifested as the increased DBSI restricted (0.03 ± 0.01 vs. control 0.01 ± 0.01, *p* = 0.29) and non-restricted (0.04 ± 0.03 vs. control 0.02 ± 0.01, *p* < 0.05) isotropic diffusion fractions, was also seen at time 1 in this particular nerve. Both DTI and DBSI measurements showed decreased λ_∥_ (DTI λ_∥_: 1.25 ± 0.32 μm^2^/ms vs. control 1.79 ± 0.12 μm^2^/ms, *p* < 0.005 and DBSI λ_∥_: 1.57 ± 0.17 μm^2^/ms vs. control 1.79 ± 0.12 μm^2^/ms, *p* < 0.005) and increased λ_⊥_ (DTI λ_⊥_: 0.22 ± 0.06 μm^2^/ms vs. control 0.16 ± 0.04 μm^2^/ms, *p* < 0.005 and DBSI λ_⊥_: 0.21 ± 0.03 μm^2^/ms vs. control 0.17 ± 0.03 μm^2^/ms, *p* = 0.16) at time 2, suggesting axonal and myelin injury, respectively [16]. Consistent with the increased DBSI restricted (0.08 ± 0.05 vs. control 0.01 ± 0.01, *p* < 0.005) and non-restricted (0.09 ± 0.06 vs. control 0.02 ± 0.01, *p* < 0.05) isotropic diffusion fractions, exaggerated DTI λ_∥_ and λ_⊥_ change at time 2 paralleled inflammatory cell infiltration and putative vasogenic edema (Fig. 2). Data from eye 1 and eye 2 were averaged at baseline, time 1 and time 2. Exaggerated changes in DTI-derived maps (Fig. 3a–c) when compared to DBSI-derived λ_∥_, λ_⊥_, and FA (Fig. 3d–f) at time 2, resulted from confounding effects from inflammation (Fig. 3g, h). For the EAE mice in this cohort, slightly increased inflammatory cell infiltration (seen as increased DBSI restricted fraction, Fig. 3h) and significant edema (as increased DBSI non-restricted fraction, Fig. 3g, *p* < 0.05) at time 1 were seen while axon and myelin damage was still absent (Fig. 3d–f). The EAE mice in this cohort developed axonal injury (Fig. 3d), mild demyelination (Fig. 3e), and significantly increased cell infiltration (Fig. 3g) and edema (Fig. 3h) at acute ON.

### DBSI detected and quantified axonal loss in the presence of optic nerve swelling

**Fig. 4:**
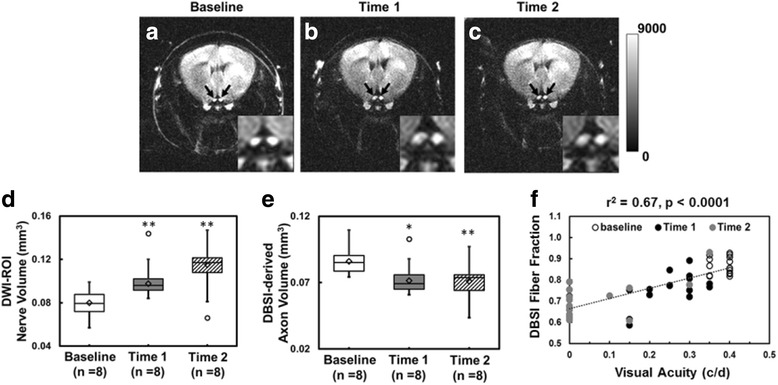
Diffusion-weighted images (DWI) were acquired using the diffusion gradient applied perpendicular to the optic nerves (*black arrows*), at baseline (**a**, before EAE induction), time 1 (**b**, onset of ON in the first eye), and time 2 (**c**, onset of ON in the second eye) from an representative EAE mouse. Optic nerve swelling was seen at time 1 and 2 caused by inflammation associated increase in cellularity and edema. Significantly increased optic nerve volume was seen after ON (**d**, *p* < 0.005). The corresponding DBSI-derived axon volume (optic nerve volume × DBSI fiber fraction) suggested a significant axonal loss in optic nerves (**e**, *p* < 0.05 and *p* < 0.005 for time 1 and 2, respectively). DBSI fiber fraction, reflecting effects of axonal loss and dilution effect of axonal density from inflammation, correlated well with visual acuity (**f**). *One asterisk* indicates *p* < 0.05. *Double asterisks* indicate *p* < 0.005

Onset of EAE ON was highly associated with inflammatory cell infiltration and putative edema, which led to optic nerve swelling in diffusion-weighted images (DWI, Fig. 4a–c). Group-average of nerve volume showed significant swelling at time 1 (0.10 ± 0.01 mm^3^ vs. control 0.08 ± 0.01 mm^3^, *p* < 0.005) and 2 (0.12 ± 0.02 mm^3^ vs. control 0.08 ± 0.01 mm^3^, *p* < 0.005, Fig. 4d). The corresponding DBSI-derived axon volume (nerve volume multiplying DBSI fiber fraction of corresponding ROI) demonstrated significant 16 and 17% axonal loss at time 1 and time 2, respectively (Fig. 4e). DBSI fiber fraction correlated well with VA measurement from baseline to time 2 (Fig. 4f).

### Immunohistochemistry of optic nerve

**Fig. 5:**
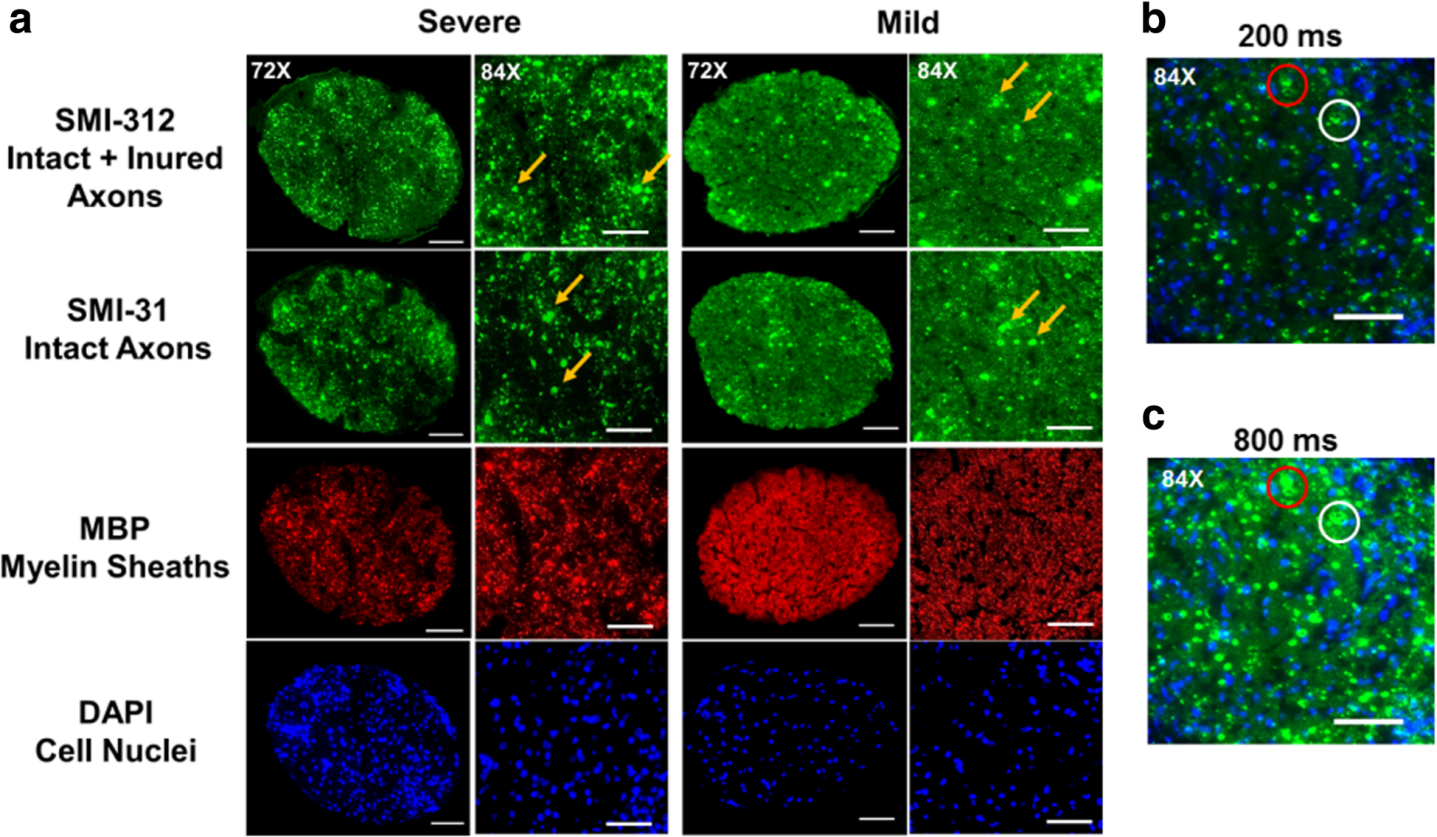
Representative ×72 immunohistochemical staining images of anti-total neurofilament (SMI-312, total axons), phosphorylated neurofilament (SMI-31, intact axon), myelin basic protein (MBP, myelin sheath), and 4′, 6-dianidino-2-phenylindole (DAPI, nuclei) from severe (**a**, column 1) and mild (**a**, column 3) optic neuritis nerves demonstrate the different degrees of tissue damages. The corresponding ×84 zoom-in images (covered ~50% of the optic nerve cross-section area) are displayed alongside ×72 images (**a**, column 2 and 4, respectively). Swollen axons, some aggregated to form enlarged staining regions (**a**, *yellow arrows*), were seen in SMI-312 and SMI-31 staining images. Axonal loss and injury (reduced SMI-312 and SMI-31 positive staining), demyelination (decreased MBP positive staining), and cell infiltration (increased density of DAPI staining) were present in optic neuritis nerves. The zoom-in ×84 DAPI and SMI-31 double-staining images from one EAE optic nerve with 200 ms (**b**) and 800 ms (**c**) exposure time revealed the multiple-axon aggregation underlying the unusually large green spots seen in the SMI-312 and SMI-31 images (**b**, **c**, *red and white circles*). *Scale bar* 50 μm

**Fig. 6:**
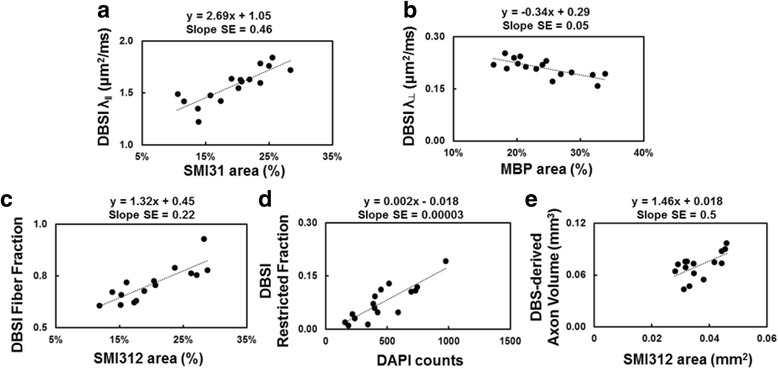
Regression of SMI-31, MBP, SMI-312, DAPI counts, and DBSI-derived λ_ǁ_ (**a**), λ_⊥_ (**b**), fiber fraction (**c**), and restricted fraction (**d**) suggested DBSI measurements were able to reflect specific pathologies in the optic nerves of EAE mice, respectively. The regression of DBSI-derived axon volume correlated with SMI-312 area (**e**, in mm^2^). In contrast to SMI-312 area estimated as ratio of positive area over the total nerve cross-sectional area (%, **c**), SMI-312 area in square millimeter reflects the extent of total axons without the dilution effect of inflammation. *SE* standard error

Post-MRI immunohistochemistry staining of optic nerves (Fig. 5) was used to assess axon (SMI-312, SMI-31) and myelin (MBP) integrity, and extent of cellularity (DAPI). The EAE optic nerves showed significant axonal swelling (yellow arrows, Fig. 5a), inflammation, and axonal injury (red and white circles, Fig. 5b, c). Both eyes from each EAE mouse was selected for the regression analyses. The regression of SMI-31, MBP, and SMI-312 area fraction and DBSI λ_∥_, DBSI λ_⊥_, DBSI fiber fraction, and DBSI restricted fraction (Fig. 6a–d) supported that DBSI indexes were able to reflect the optic-nerve pathologies. Similarly, DBSI-derived axon volume was able to reflect SMI-312 area (Fig. 6e).

## Discussion

We examined optic nerve pathology in EAE mice at the onset of the ON when VA impairment was observed (Fig. 1). Optic neuritis in EAE-affected mice, much like in MS, is heterogeneous in pathology with a mixture of axonal injury, demyelination, cellular inflammation, and edema (Fig. 5) [22, 29–31]. We employed DBSI to monitor the evolving optic nerve pathology in EAE mice with ON by distinguishing and quantifying inflammation, demyelination, and axonal injury/loss simultaneously (Figs. 3, 4, and 6). DBSI parameters suggested the presence of prominent inflammation-associated increase in cellularity and edema at the onset of ON (Fig. 3g, h), consistent with the postmortem immunohistochemistry findings (Figs. 5 and 6). These pathological components not only contributed to the impaired visual function clinically but also confounded interpretation of DTI derived axonal injury and demyelination metrics (Fig. 3a–c).

Current MRI diagnostic approaches fail to accurately assess the progression of MS. Advanced MRI measures such as quantitative relaxation, diffusion, and magnetization transfer imaging provide more information than conventional MRI but unfortunately cannot distinguish between reversible and irreversible pathologies. Imaging markers sensitive and specific to axonal loss, which is thought to be irreversible, would provide the critical tools needed for assessing MS progression. The advent of optical coherence tomography (OCT) has enabled the quantification of neuronal (ganglion cell layer/inner plexiform layer, GCL + IPL) and axonal (retinal nerve fiber layer, RNFL) loss in the visual system allowing the direct correlation of structure with function [32–34]. In MS patients with or without history of clinical optic neuritis, GCL + IPL and RNFL thinning can be observed [35, 36]. Interestingly, OCT-detected RNFL thinning has also been reported to correlate with brain atrophy [37–41]. The portions of the anterior visual pathway measured using OCT have thus been considered to reflect the more global central nervous system (CNS) integrity; OCT has increasingly been suggested as an outcome measure in MS [42–45].

However, OCT is not as useful in the presence of early acute inflammation, due to the confounding presence of acute cell infiltration and vasogenic edema [46]. The posterior visual pathway (optic nerves/tracts/radiations) is not directly visualized by OCT due to the limited penetration of the technique. Moreover, the ability of OCT-detected intraocular pathologies to represent CNS pathologies outside of the visual system is indirect and imperfect in the individual patient. Thus, imaging biomarkers that can interrogate the entire CNS white matter, and distinguish and quantify different components of pathology without succumbing to their confounding interferences are greatly needed in MS. DBSI fiber fraction estimated axonal density of the optic nerve including the dilution effect of inflammation (optic nerve swelling on DWI, Fig. 4a–c) in each voxel. The correlation of VA and DBSI fiber fraction indicated that visual function was affected by inflammation (reversible) and axonal loss (irreversible, Fig. 4d). The recovery of visual function independent of initial visual loss in MS patients with optic neuritis may suggest the irreversible axonal loss is below the threshold of permanent vision loss [10, 47].

We contend that DBSI could provide the unmet needs in MS and neurological disorders in general by presenting specific pathological metrics to quantitatively reflect axonal injury, demyelination, inflammation, and axonal loss. A longitudinal DBSI measurement could assess the effectiveness of anti-inflammatory therapies on axonal preservation by longitudinally assessing axonal pathologies in real time. The axonal loss in this cohort of EAE mice occurred early (Fig. 6e). Since axonal integrity plays a crucial role in neurological disability [48, 49], longitudinal measurements of DBSI-derived axonal volume could potentially quantify the rate of irreversible axonal loss and serve as a biomarker of MS progression preceding detectable clinical symptoms.

## Conclusions

Our findings support the notion that axonal loss could occur early in EAE mice. Diffusion basis spectrum imaging detected pathologies in the posterior visual pathway unreachable by optical coherence tomography and without confounding inflammation induced optic nerve swelling. Diffusion basis spectrum imaging could thus decipher the interrelationship among various pathological components and the role each plays in disease progression. Quantification of the rate of axonal loss could potentially serve as the biomarker to predict treatment outcome and to determine when progressive disease starts.

## Abbreviations

CNS: Central nervous system
DBSI: Diffusion basis spectrum imaging
DTI: Diffusion tensor imaging
EAE: Experimental autoimmune encephalomyelitis
GCL + IPL: Ganglion cell layer/inner plexiform layer
MS: Multiple sclerosis
OCT: Optical coherence tomography
ON: Optic neuritis
PBS: Phosphate-buffered saline
PFA: Paraformaldehyde
RNFL: Retinal nerve fiber layer
VA: Visual acuity
